# College and Hospital Notes

**Published:** 1911-07

**Authors:** 


### COLLEGE AND HOSPITAL NOTES

THE day camp for tuberculosis at Main and LaSalle streets, Buffalo, was opened the latter part of May with 30 patients. The camp is composed of a dining tent and a shelter; eight tents for patients who remain at the camp day and night, a tent for the nurses and one for the physician in charge. Two of the tents are the gifts of the Coterie and Daffodil branches of the Sunshine Society. A three compartment tent has been donated by Mrs. Daniel Good. Dr. George J. Eckel is physician in charge and the nurses are Miss Phyllis Wood and Miss Grace Holmes.

Mrs. A. J. Elias is chairman of the committee on house and grounds; Mrs. J. H. Pryor, of the steward's committee; Mrs. D. Maxon Estee, tents; Mrs. Charles E. Baker, furnishings and house linen; Mrs. A. W. Thorn, flowers. Mrs. Baker is also in charge of the philanthropic committee. Applications have been received from 150 patients and within a month it is expected there will be 150 patients needing the services of the camp. During the coming month 75 patients will be cared for.

---

THE supervisors of Erie county have voted against separation of the tuberculosis hospital from the county hospital by a vote of 36 to 13. Prior to the taking of the vote the supervisors were addressed in favor of separation by Drs. DeLancey Rochester and R. R. Ross; John R. Shillady, Charles Stevens and John Coleman who made it plain that, while the administrations of the keeper of the county poorhouse and the superintendent of the county hospital were not criticised, it was for the betterment of the tuberculosis patients that the hospital in which they were housed should be under the control of a board of managers. The salient feature of the discussion was the declaration of supervisor Emery, of East Aurora, that medical politics was behind the plan of separation. He was willing that tubercular patients should remain as poorhouse inmates.

---

BY the will of Ada M. Kenyon, probated in May, the bulk of her estate valued at about $5,000 was given to the Buffalo Homeopathic Hospital as an endowment fund for a free bed to be used for the women teachers of the public schools of Buffalo.

---

THE new Homeopathic Hospital building at Lafayette and Linwood avenues, Buffalo, was formally opened for public inspection June 3, the reception lasting through the afternoon and evening. The building was elaborately decorated with American beauty roses, carnations and peonies, donated by the Junior Board of the hospital. The Willing Workers served refreshments in the solarium, and in the board room Mrs. Edward A. Eisle and her committee served tea. The charter members of the board of managers was received by Mrs. Joseph Tottenham Cook and the reception committee, Mrs. W. H. Crosby, Miss H. E. Montgomery, Mrs. E. J. Barcalo, and Miss Edith Donaldson.

The hospital was dedicated on Sunday, June 5. Visitors were received by a reception committee of 100 women interested in the institution. The exercises were brief, Dr. A. V. V. Raymond making the principal address in which he referred to the campaign during which public subscription provided sufficient funds to make the hospital a reality. Dr. Raymond was followed by the Rev. S. V. V. Holmes and the Rev. Charles A. Jessup. The musical portion of the exercises was furnished by the choir boys of Ascension Church. Following the dedicatory exercises a brief ritual service was held on each floor of the building.

Patients were removed from the old hospital building to the new on Wednesday, June 7.

The hospital has been built on the most modern plans and with a view to the comfort of the patients.

In the administration building are a library, waiting room, offices for the superintendent of nurses, the assistant superintendent, the doctors, consulting-rooms, and a large board and lecture room.

The ward floor is across the corridor, which leads one into the regular hospital pavilion. The ground floor of this pavilion is arranged in wards, medical and surgical, for men and women. The babies' wards are on this floor. The wards open upon balconies, on a level with the ward floors, so that the beds of the patients can be pushed outside through the broad doors, and the patient may have fresh air without being obliged to move.

Each floor has its separate supply-room, serving- room, closets, and the like. The serving-rooms are equipped with a hot-water table, where the dishes sent up from the basement kitchen are kept hot; large pantries, ice boxes and gas stoves.

On the second floor are the private rooms. Some are in suite of bedroom, sittingroom and bath, and the equipment of all the rooms is complete. The superintendent of the training school has a suite on this floor, bedroom, sitting-room and bathroom. Another suite is set apart for the diet nurse. A laboratory is on this floor. Two surgeries are on the third floor, with proper adjoining rooms for the doctors, the patients and the attendants. There are also on this floor two wards reserved for eye patients.

The maternity wards are equipped with two operating rooms. The internes have six bedrooms and a sitting room on the fourth floor. The fifth floor is a roof garden.

In the basement are dining rooms and bathrooms for all kinds of therapeutic baths. The big main kitchen is here, two *x*-ray rooms, with dark rooms attached, a model diet kitchen, supply-rooms of all sorts and a department, where accident and other emergency cases are disposed of. There are accommodations for 140 patients in the new hospital.

---

THE right wing of the German Hospital, Buffalo, formerly occupied as nurses' quarters, has been made into three private and semi-private rooms, which have been furnished by Mr. William F. Kastings, Henry vom Berge and Dr. J. G. Meidenbauer. Mr. Kasting's room is furnished in mahogany and the room equipped by Mr. Vom Berge is fitted up in colonial style. Dr. Meidenbauer's room contains four beds and is a semi-private ward. The nurses of the hospital now are living in two houses adjoining the hospital which have been purchased and made into a complete nurses' home. The new rooms were opened for inspection on June 5 and hundreds of friends of the institution visited the hospital and were loud in their praises of the improvement. Linen for the new rooms has been provided by the ladies' sewing circle.

---

THE social service committee of the Children's Hospital has made arrangements for the care of sixteen children during the summer. The committee will pay for the services of a trained nurse and assistant and fill Saint Margaret's cottage with its proteges who are either convalescing from illnesses or being prepared for operation at the hospital. On June 17 motor cars, provided by Mrs. Frank [illegible] ear, Mrs. Henry O. Smith, Mrs. Laurence H. Gardner and Mrs. O. E. Foster took the children to the station where they were met by five members of the committee who took them to the Fresh Air Mission to remain until they are well enough to return to their homes or the hospital, after which their places will be taken by others. This work will be carried on all summer.

---

THE social service committee of the Children's Hospital at the June meeting received reports on sixteen new cases and nine old ones. Miss Doyle reported on the ward visits and the study and work with the boys and girls. An especial feature of the work of the committee is the Sunday afternoon visits when Miss Dorothy Gerrans sings or Miss Doyle reads to the little patients.

---

A GIFT of $20,000, to be used in the study of diseases at the Yale Medical School, as announced by Secretary Anson Phelps

Stokes, Jr., of Yale University, has been given by Francis E. Loomis, of the class of 1864. Further gifts of $10,000 toward the endowment of the university clinic and to the Peruvian exploration fund for the Yale expedition, under Assistant Professor Hiram Bingham, have been announced.

---

Mr. John R. Shillady, secretary of the Association for the relief and control of tuberculosis has given to the daily newspapers an interview in which he shows the needs of the day camp and the necessity for prompt support from the public. After speaking of the number of applications in the hands of Dr. George Eckel, the physician in charge of the camp, which, by the way, far exceeds the present capacity, Mr. Shillady said:

"In a week or two we expect to have 75 patients on the grounds. That means that we shall have to raise about $4,000 more than we now have for the day camp part of our work alone. Last year we had almost enough money from the sale of the red cross Christmas stamps to run the camp; this year we are about $4,000 short. We simply cannot refuse to care for the unfortunates who depend on us. For one thing, it will cost about $750 for nurses. Last year these were donated to us by the District Nursing Association, but the demands upon them have been so great they are unable to support the nurses this year. Then the city makes no provision for taking care of advanced consumptives in its hospitals, as it does other cases of chronic diseases, so our association is the only resource these poor unfortunates have. They won't go to the Erie county hospital. A great many of them when appealed to say they do not want to die in a poorhouse or to have it said that their father or mother was in an almshouse. That hospital, of course, does have to bear that stigma.

"We tried to have the supervisors place the tuberculosis pavilion at the county hospital under a board of managers, so as to separate it from the almshouse, but they refused. As long as that hospital is run under the system it is now run, with the responsibility divided between the keeper of the almshouse, medical superintendent, visiting staff of physicians and the almshouse and hospital committee of the supervisors, we can never expect good work in the care and treatment of tuberculosis patients.

"It is up to the city of Buffalo at the present time to make some proper provision for these advanced consumptives. They have been allowed to die in their homes long enough. I expect the association will take up the matter of urging upon the city the erection of a tuberculosis hospital for 250 advanced cases."

The Columbus Hospital, of Buffalo, celebrated the third anniversary of its founding, Thursday evening, June 15, and also graduated it first class of nurses. The hospital was handsomely decorated with flowers and with the United States and Italian flags. Many of Buffalo's representative citizens were present, professional and otherwise. The members of the graduating class were Miss Jennie T. Vilia of Farnham and Miss Mary J. Brynes of Buffalo.

The exercises were held in one of the wards on the ground floor. Dr. James W. Putman addressed the graduates. He said in part: "I am grateful for the honor of being called upon to address the first graduation class of the Columbus Hospital, which is dedicated to the cause of humanity and engaged in the noble work of relieving the suffering of the sick and afflicted. I am pleased at this opportunity to pay tribute to the genius of the man, Dr. Borzilleri, who saw the need of an institution, such as this, and who has reared a hospital dedicated to scientific attainment and fully abreast, in every particular, with the times. It has a cosmopolitan staff of men, standing foremost in the respective branches of the profession they represent, and imbued with the earnestness and enthusiasm of its founder. . Started three years ago, this institution has not solicited and has not yet received aid from any source, but, nevertheless, has won the approbation of the medical profession and the public, and its graduates will go forth into the world with diplomas, meaning fully as much as those issued by older and better known hospitals. These diplomas will guarantee you recognition and protection wherever you may go and the support of chivalrous humanity no matter whither your steps may lead you."

Professor M. Cabone, Italian consul, spoke to the graduates in Italian, and presented the insignia of the hospital to them in the shape of beautiful gold pins bearing the initials "B. C. H." entwined about the red cross. Father Angelo Strazzoni of Saint Anthony's Church presented the diplomas to the graduates with words of counsel, and the rest of the evening was devoted to festivities and to an inspection of the hospital which has been enlarged recently by the addition of a new surgical ward.

---

The managers of the Children's Hospital, Buffalo, have adopted plans for the nurses' home to be built on Bryant street next to the hospital. It is hoped to finish the structure this autumn. Mrs. Frederick L. Pratt, Mrs. Charles L. Gurney, Mrs. Folwell and Mrs. Pardee are visitors for the month.

---

The commencement exercises of the training school for nurses of the Children's Hospital, Buffalo, were held Tuesday evening,

June 20, in the gymnasium of the Elmwood school. The room was handsomely decorated and in the center of the stage was a great basket of white roses, the gift of Mrs. William Hamlin to the class of 1911.

The junior class entered first, forming an aisle through which the seniors marched. The members of the hospital staff were on the stage, also the Reverend Dr. Charles A. Jessup, rector of the Church of the Ascension, who offered prayer and made the commencement address:

Dr. H. G. Matzinger presented the hospital pins to the graduates and Dr. Frederick J. Parmenter read the Florence Nightingale pledge.

Two songs, by Miss Harriet Keating, preceded the presentation of diplomas to the graduates by Dr. Charles Sumner Jones.

Following the commencement was a reception and dance in honor of the graduating class. A buffet supper was served in one of the schoolrooms from a table bearing a centerpiece of white carnations, surrounded by small bunches of daisies and trailing ferns.

The members of the class of 1911, the largest ever graduated from the Children's Hospital, were as follows: The Misses Maude Pierce, Mae Clark, Hazel Hotson, Mary Earon, Rose Barry, Harriet Rumsey, Rose Gibson, Eva Hamilton and Anna Van Valkenburg.

